# Inhibition of natural killer (nk) cell cytotoxicity by interleukin-6: implications for the pathogenesis of macrophage activation syndrome

**DOI:** 10.1186/1546-0096-12-S1-P56

**Published:** 2014-09-17

**Authors:** Loredana Cifaldi, Giusi Prencipe, Ivan Caiello, Claudia Bracaglia, Raffaele Strippoli, Fabrizio De Benedetti

**Affiliations:** 1 Paediatric Haematology/Oncology, IRCCS Ospedale Pediatrico Bambino Gesù, Italy; 2 Rheumatology, IRCCS Ospedale Pediatrico Bambino Gesù, Italy; 3 Cellular Biotechnologies and Haematology, Sapienza Rome University, Rome, Italy

## Introduction

MAS occurs frequently in patients with active sJIA and because of the similarities with HLH is classified among secondary HLH. Systemic juvenile idiopathic arthritis (s-JIA) is characterized by high levels of Interleukin-6 (IL-6). Impairment of natural killer (NK) cell function and decrease perforin expression have also been reported in sJIA.

## Objectives

Aim of this study was to evaluate the effect of IL-6 on NK cell cytotoxic function.

## Methods

Following in vivo treatment with poly(I:C), splenic NK cell cytotoxic activity from wild tipe (WT) or IL-6 transgenic (IL-6TG) mice was evaluated using the chromium51 release assay. NK cell number, perforin, GranzymeB, CD69 and CD107a expression were evaluated by flow cytometric analysis. Human polyclonal NK cells were expanded from peripheral blood mononuclear cells (PBMCs) in co-cultures with the feeder cell line RPMI8866 in the presence of tocilizumab, an IL-6 receptor blocker, or isotype control. IL-6 production in the supernatants of human polyclonal NK cells was measured by ELISA. PBMCs from healthy donors were treated with IL-6. NK cell cytotoxic activity, Perforin and CD107a expression were evaluated as above.

## Results

Following poly(I:C) administration, in vivo generation of splenic NK cell cytotoxicity was markedly reduced in IL-6TG compared to WT mice. In IL6TG mice number of NK cells, number of CD69+ NK cells and degranulation were comparable to WT mice. Defective expression of both perforin and GranzymeB were found in NK cells from IL-6TG mice. High levels of IL-6 were found in the supernatants of human polyclonal NK cells. Neutralizing IL-6 with tocilizumab in co-cultures of human PBMCs increased human NK cell cytotoxicity and perforin expression. Addition of IL-6 to human PBMCs decreased perforin expression in NK cells.

## Conclusion

Both in vivo in mice and in vitro in humans, IL-6 inhibits NK cytotoxicity down-regulating perforin expression. In patients with prominent inflammatory response, such as JIA, high levels of IL-6 may contribute to the induction of MAS also by inhibiting cytotoxicity inducing a defect similar to that of primary HLH.

## Disclosure of interest

None declared.

